# Deriving Weight From Big Data: Comparison of Body Weight Measurement–Cleaning Algorithms

**DOI:** 10.2196/30328

**Published:** 2022-03-09

**Authors:** Richard Evans, Jennifer Burns, Laura Damschroder, Ann Annis, Michelle B Freitag, Susan Raffa, Wyndy Wiitala

**Affiliations:** 1 Center for Clinical Management Research Veterans Health Administration Ann Arbor, MI United States; 2 College of Nursing Michigan State University Lansing, MI United States; 3 National Center for Health Promotion and Disease Prevention Veterans Health Administration Durham, NC United States; 4 Department of Psychiatry & Behavioral Sciences Duke University School of Medicine Durham, NC United States

**Keywords:** veterans, weight, algorithms, obesity, measurement, electronic health record

## Abstract

**Background:**

Patient body weight is a frequently used measure in biomedical studies, yet there are no standard methods for processing and cleaning weight data. Conflicting documentation on constructing body weight measurements presents challenges for research and program evaluation.

**Objective:**

In this study, we aim to describe and compare methods for extracting and cleaning weight data from electronic health record databases to develop guidelines for standardized approaches that promote reproducibility.

**Methods:**

We conducted a systematic review of studies published from 2008 to 2018 that used Veterans Health Administration electronic health record weight data and documented the algorithms for constructing patient weight. We applied these algorithms to a cohort of veterans with at least one primary care visit in 2016. The resulting weight measures were compared at the patient and site levels.

**Results:**

We identified 496 studies and included 62 (12.5%) that used weight as an outcome. Approximately 48% (27/62) included a replicable algorithm. Algorithms varied from cutoffs of implausible weights to complex models using measures within patients over time. We found differences in the number of weight values after applying the algorithms (71,961/1,175,995, 6.12% to 1,175,177/1,175,995, 99.93% of raw data) but little difference in average weights across methods (93.3, SD 21.0 kg to 94.8, SD 21.8 kg). The percentage of patients with at least 5% weight loss over 1 year ranged from 9.37% (4933/52,642) to 13.99% (3355/23,987).

**Conclusions:**

Contrasting algorithms provide similar results and, in some cases, the results are not different from using raw, unprocessed data despite algorithm complexity. Studies using point estimates of weight may benefit from a simple cleaning rule based on cutoffs of implausible values; however, research questions involving weight trajectories and other, more complex scenarios may benefit from a more nuanced algorithm that considers all available weight data.

## Introduction

### Background

The use of electronic health records (EHRs) by health care systems has rapidly increased during the last 2 decades [1], making vast amounts of clinical information available for use in research and evaluation efforts [2,3]. However, there are issues associated with using EHR data, including a lack of control over data definitions and data collection processes [4] as well as methodological challenges associated with processing and transforming raw, messy EHR [5] data into research-ready data that can be meaningfully used for research and evaluation [6]. For these reasons, many have called for increased transparency regarding data cleaning efforts, methods to assess EHR data quality [7], and increased reporting and sharing of methods for selecting clinical codes [8,9].

Obesity is associated with increased risk of a wide range of medical problems, including diabetes, hypertension, high blood cholesterol, cardiovascular events, bone and joint problems, and sleep apnea [10]. Clinicians frequently advise patients to lose weight to help prevent or delay the onset of chronic disease [11]. Accordingly, obesity is a major public health challenge for the United States; compared with patients of normal weight, patients with obesity have higher inpatient costs, more outpatient visits and costs, and more spending on prescription drugs [12]. Thus, patient weight represents a frequently used measure for many researchers and evaluators. It may be included as a risk factor in studies seeking to predict adverse medical events, as a covariate in studies that seek to adjust for the effect of baseline weight when examining the association between another variable (eg, treatment) and an outcome, or as an outcome in studies examining the effects of a measure (eg, intervention) on patient weight or weight change over time.

Despite being a common clinical measure, there is no standard for processing and cleaning EHR weight data for use in research and evaluation studies. Researchers are often left to select and replicate a method described by others or develop their own algorithms to define weight measures for analyses, resulting in many different definitions in the published literature [13]. These definitions range from simple cutoffs for implausible values to more computationally complex algorithms requiring significant coding and processing capacity, as well as difficulties in replicating for other studies. Furthermore, it is unknown how resulting weight measures may vary based on how researchers process and clean the data; subsequently, the impact of algorithm choice on results and research findings is also unknown.

### Study Objective

The objectives of this study include (1) comparing algorithms for extracting and processing clinical weight measures from EHR databases and (2) providing recommendations for the use of algorithms. We used measures of patient weight from the Corporate Data Warehouse (CDW) of the Veterans Health Administration (VHA) to accomplish these objectives. The VHA includes a network of medical centers that rely on a system-wide integrated EHR system. Patient data are extracted from EHR records nightly and uploaded to a centralized CDW, which comprises relational data tables that can be accessed by data analysts, including researchers. Users extract data from the CDW and typically perform simple data checks to verify accuracy. More complex algorithms may be used, especially in research; for example, to ensure that the amount of missing data does not exceed a prespecified threshold [14].

## Methods

### Cohort and Data Sources

We included cohorts of VHA patients based on two calendar year periods: 2008 and 2016. Previous work suggests that data quality for some CDW data fields has improved over time in terms of cleanliness and data capture [15,16]. Thus, selecting 2 time points allowed us to compare the quality and quantity of data between these time points. For each year, we randomly sampled 100,000 patients aged ≥18 years with at least one primary care visit (VHA Stop Code 323) during the cohort year, with the first primary care visit serving as the index date. There were no restrictions on facility or region; thus, our cohorts represent a national sample. We excluded patients with any International Classification of Diseases, 9th or 10th revision codes, or Current Procedural Terminology codes for pregnancy within 2 years before and 2 years after the index date, which we henceforth refer to as the *collection period*. Our detailed approach is described in Multimedia Appendix 1 [17-28].

We collected all weight and height measurements from the CDW vital sign table during the collection period. If a patient had more than one height measurement during the 4 years, we used the modal value to determine a single measure of height for each patient. In the event that an individual only had 2 recorded height values, the last value was chosen when height was arranged in ascending order by collection date. We calculated BMI by dividing weight in kilograms by height in meters squared. All weight and height data were cleansed of any nonnumeric characters, converting commas to decimals where appropriate.

### Weight-Cleaning Algorithms

Previously, our team conducted a systematic literature review to identify studies that used patient weight outcome measures from the VHA CDW [13]. We identified 39 published studies that used the CDW to define patient weight outcomes. Of the 39 studies, 33 (85%) [17-49] included a weight-cleaning algorithm that could be implemented and replicated in this study. In this paper, we present 12 algorithms [17-28] representing the breadth of methods used in cleaning body weight measurements and provide details about the remaining algorithms in Multimedia Appendix 1 and in our GitHub repository [50].

For comparison, we divided the 12 algorithms into two conceptual groups: (1) those that included all weight measurements during a specified time frame and (2) those that were period-specific. A brief description of the key differences between algorithms by group is shown in Table 1. Period-specific algorithms were those that selected *baseline, 6-month, and 12-month* periods and included weight measurements during specified windows around those dates. Note that not all algorithms fit exactly into these groups. For instance, we classified the algorithm used in the study by Noël et al [27] as a *period-specific* algorithm, as it is based on fiscal quarters but uses all data within each quarter to define median weights. Similarly, the algorithm by Jackson et al [21] involves taking the arithmetic mean of all weight measurements collected between arbitrarily chosen time points.

All algorithms were recreated from the methods sections described in the relevant publications and translated into pseudocode and then into R (version 3.6.1; R Foundation for Statistical Computing) or SAS (version 9.4; SAS Institute) code (Multimedia Appendix 1, section 2, and web-based supplemental materials [50]).

**Table 1:** Conceptual description of main exclusions after applying each algorithma.

Conceptual group		Exclusions based on algorithm
**All weight measures**		
	Buta et al [18]	Patients with ≤1 weight value BMI <11 or >70
	Chan and Raffa [19]	Weights <23 kg or >340 kg Weights >3 SD from mean
	Maguen et al [26]	Weights <32 kg or >318 kg Weights where the absolute value of conditional residual from linear mixed model ≥10
	Breland et al [17]	Weights <34 kg or >318 kg Weight values that fell outside of specific ratios calculated within patients over time
	Maciejewski et al [25]	Weight values associated with large SDs calculated on a rolling basis
	Littman et al [24]	Weights <34 kg or >272 kg Weights where difference from mean >SD Weights where SD was >10% of the mean
**Period-specific**		
	Rosenberger et al [28]	Patients with <K number of weight measures; K chosen by researcher Weights outside of 6-month time points
	Noël et al [27]	Weights ≤32 kg or ≥318 kg Patients with too few values to compute median within fiscal quarters
	Kazerooni and Lim [23]	Weights outside of windows around 3 periods Patients missing data in any of the 3 periods
	Jackson et al [21]	Weights <34 kg or >318 kg Weights outside of 90-day window of each time point
	Goodrich et al [20]	Weights <36 kg or >227 kg Patients with >45 kg change between periods (baseline and 6 and 12 months) Weights outside of 30-day window of each time point
	Janney et al [22]	Weights <41 kg or >272 kg at baseline Weights outside of 30-day window of baseline and 60-day window of 6- and 12-month period Weights resulting in >45 kg change during study

^a^Details of each algorithm, including code, excerpts from published methods, and pseudocode, can be found in Multimedia Appendix 1, section 2, and the project GitHub [50].

### Methods to Compare Algorithms

#### Descriptive Statistics

All algorithms were applied to the data for both cohorts and compared based on descriptive statistics, including the number of weight measures and patients retained and the mean, SD, median, and range of weight values. For comparison, we also included descriptive statistics based on the raw, unprocessed weight data during the study time frame.

#### Weight as a Predictor

Weight is often used as a risk factor or covariate in statistical models to predict health outcomes. We present an example showing the association between baseline weight and *new-onset* diabetes to compare algorithms in this context. For this analysis, we excluded patients with diabetes before the study index date and we defined new-onset diabetes as the presence of 2 or more diabetes diagnosis codes after the patient’s index date. To create baseline weight measures for each patient, all 12 algorithms were first applied to each cohort, then weight measurements were collected given a 60-day window on or before the index date (ie, 30 days before to 30 days after the index date). The resulting baseline weight measure was the measurement that occurred on the closest day to the index date after cleaning the weight data. We then used 13 distinct logistic regression models to obtain odds ratios (ORs) for the effect of patient weight on new-onset diabetes.

#### Weight Change

A common metric used in weight loss evaluation studies involves *weight loss ≥5%*, where weight change is assessed over a 1-year period [11]. We applied each algorithm to our cohorts to compare algorithms on this metric. After cleaning the weight data, we used a 60-day window to define initial weight values and included the weight measurement taken on the closest day to the index date. To define the 1-year follow-up weight, we again used a 60-day window around the date 1 year after the baseline, keeping the closest weight measurement. In addition, using the same procedure outlined above, we computed *weight gain ≥5%* in a 1-year period.

#### Longitudinal Weight Trajectory

Weight is frequently measured, often resulting in several weight measures per patient over time. Researchers may be interested in assessing weight trajectories within patients over time and potentially classifying patients according to their trajectory or examining whether types of patients respond differentially to interventions. Algorithm choice may affect the trajectory of individuals and their measurements collected over time, especially for algorithms that severely reduce the number of measurements left to analyze. Instead of aggregating patient weight over a specific period, studies analyzing weight measures within patients over time use repeated-measure designs such as (generalized) linear mixed models (LMMs), analysis of variance, or analysis of covariance for estimation. To compare algorithms in this context, we used a latent class LMM that assumes the population is heterogeneous and composed of some selected number of latent classes characterized by specific trajectories.

The latent class mixed models implemented through the R package *lcmm* (package version 1.8.1) [51] exhibited poor or slow convergence characteristics as the sample size increased; thus, a random sample of 1000 individuals from each cohort was used for model development. The same random sample was processed by each of the 12 algorithms and evaluated using the same latent class mixed model.

#### Facility-Level Metric

Researchers and evaluators are often interested in comparing facilities according to the percentage of patients who meet a metric of interest. To examine this application, we calculated the percentage of patients with 1-year *weight loss ≥5%* and *weight gain ≥5%* at each facility using the raw data and each of the 12 algorithms. Although these types of comparisons may often be risk-adjusted, our objective was only to understand the impact of algorithm choice on calculated facility-level metrics; therefore, we examined unadjusted facility rates. We rank-ordered facilities separately based on the percentage of patients with weight loss of ≥5% and weight gain of ≥5%. We then compared the differences in the percentage of patients based on each algorithm, grouping by those that used all data and period-specific algorithms.

## Results

### Sample Descriptive Statistics

Both cohorts included approximately 100,000 patients (n=98,786 in 2008 and n=99,958 in 2016; Multimedia Appendix 1, Table S2). Patients were excluded if they had no weight measurements or were pregnant during the data collection period.

Using the raw data from the 2016 cohort, each veteran had a mean of 12.2 (SD 24.9) weights recorded over the 4-year collection period, and 1 patient had 4981 measurements (web-based supplement [50]). Approximately 5.29% (5291/99,958) of veterans had only 1 weight measurement recorded. Before applying any cleaning rules, the data included 1,175,995 total weight measurements. Between 2008 and 2016, the average weight increased by approximately 2.3 kg (91.9-94.3 kg), with a 1-point increase in SD (21.6-22.0 kg; Multimedia Appendix 1, Table S3). The number of weights recorded did not differ between the 2008 and 2016 cohorts and had similar overall distributions.

Aside from the difference in average weight between the 2 cohorts, the results did not reveal major differences in the number of weight measurements per patient or weight distributions. Therefore, the remainder of the results will focus on the 2016 cohort. The results from the 2008 cohort are included in Multimedia Appendix 1.

### Algorithm Descriptive Statistics

Descriptive statistics for the raw data and each of the 12 algorithms are shown in Table 2. After applying each algorithm to the raw data, all but 2 retained >90% of the patients—Kazerooni and Lim [23] retained approximately 24% (23,987/99,958) of patients, and Rosenberger et al [28] retained 63.43% (63,405/99,958). The mean and SD varied little between algorithm types, ranging from 93 to 95 kg (range 20.6-21.9 kg).

**Table 2:** Weight processing by algorithm and type of algorithm.

Item		Patients retained, n (% of raw weights)	Weight measurements retained, n (% of raw weights)	Weight (kg), mean (SD; range)	Weight (kg), median (IQR)
Raw weights		99,958 (100)	1,175,995 (100)	94.3 (22.0; 0-674.0)	91.8 (27.4)
**Algorithms that used all data**					
	Buta et al [18]	90,159 (90.2)	1,131,996 (96.3)	94.3 (21.9; 12.3-111.1)	91.9 (27.3)
	Chan and Raffa [19]	96,132 (96.2)	1,170,114 (99.5)	94.3 (21.9; 24.5-330.0)	91.8 (27.4)
	Maguen et al [26]	98,352 (98.4)	1,037,293 (88.2)	93.3 (21.0; 31.9-245.4)	91.0 (26.4)
	Breland et al [17]	99,958 (100)	1,175,177 (99.9)	94.3 (21.9; 34.0-315.0)	91.8 (27.4)
	Maciejewski et al [25]	99,958 (100)	1,146,995 (97.5)	94.4 (21.8; 28.1-247.7)	91.9 (27.2)
	Littman et al [24]	96,130 (96.2)	1,161,661 (98.8)	94.3 (21.8; 34.0-247.7)	91.9 (27.2)
**Period-specific algorithms**					
	Rosenberger et al [28]	63,405 (63.4)	227,215 (19.3)	94.3 (21.0; 0-596.2)	92.0 (26.3)
	Kazerooni and Lim [23]	23,987 (24)	71,961 (6.1)	94.8 (21.8; 0-559.6)	92.5 (27.2)
	Goodrich et al [20]	95,748 (95.8)	199,830 (17)	93.5 (20.6; 36.3-226.8)	91.2 (25.7)
	Janney et al [22]	95,742 (95.8)	199,830 (17)	93.5 (20.6; 35.6-247.7)	91.2 (25.7)
	Jackson et al [21]^a^	96,559 (96.6)	251,501 (21.4)	93.6 (20.6; 27.4-259.0)	91.2 (25.9)
	Noël et al [27]^a^	99,958 (100)	683,008 (58.1)	94.0 (20.9; 31.8-267.1)	91.6 (26.1)

^a^These algorithms differ from the other period-specific algorithms as they first use all available data and then proceed to aggregate measures by the mean or median within select periods.

The raw, unprocessed data contained implausible values ranging from 0 kg to 674 kg. Although most algorithms involved removing outlying values—often as the first step—some did not. Most notably, data processed by two of the algorithms (Kazerooni and Lim [23] and Rosenberger et al [28]) maintained weight values from 0 kg to >454 kg (see Table 1 for algorithm descriptions).

Algorithms designed to use all available weights retained a bulk of the measurements (1,037,293/1,175,995, 88.21% to 1,175,177/1,175,995, 99.93%) and resulted in a similar average weight (mean 93.3-94.4, SD 21.0-22.0 kg). The SD did not decrease after applying the algorithms except for the algorithm by Maguen et al [26], which retained 88.21% (1,037,293/1,175,995) of the measurements and resulted in a slightly lower average weight and SD (mean 93.3 kg, SD 21.0 kg).

For the period-specific algorithms, only 1 retained >50% of the raw weight measurements (Noël et al [27] maintained 683,008/1,175,995, 58.08% of the available data), yet the average weight and SDs differed little between algorithms. The Kazerooni and Lim [23] algorithm resulted in higher average and median weights (mean 94.8 kg, SD 21.8 kg, median 92.5 kg). It is important to note that the algorithms designed by Kazerooni and Lim [23] and Noël et al [27] first use all available data and then proceed to aggregate measures by the mean or median within select periods. Thus, they differ in approach from the other period-specific algorithms, which first define periods and then extract weight measures during windows around those periods.

**Figure 1:**
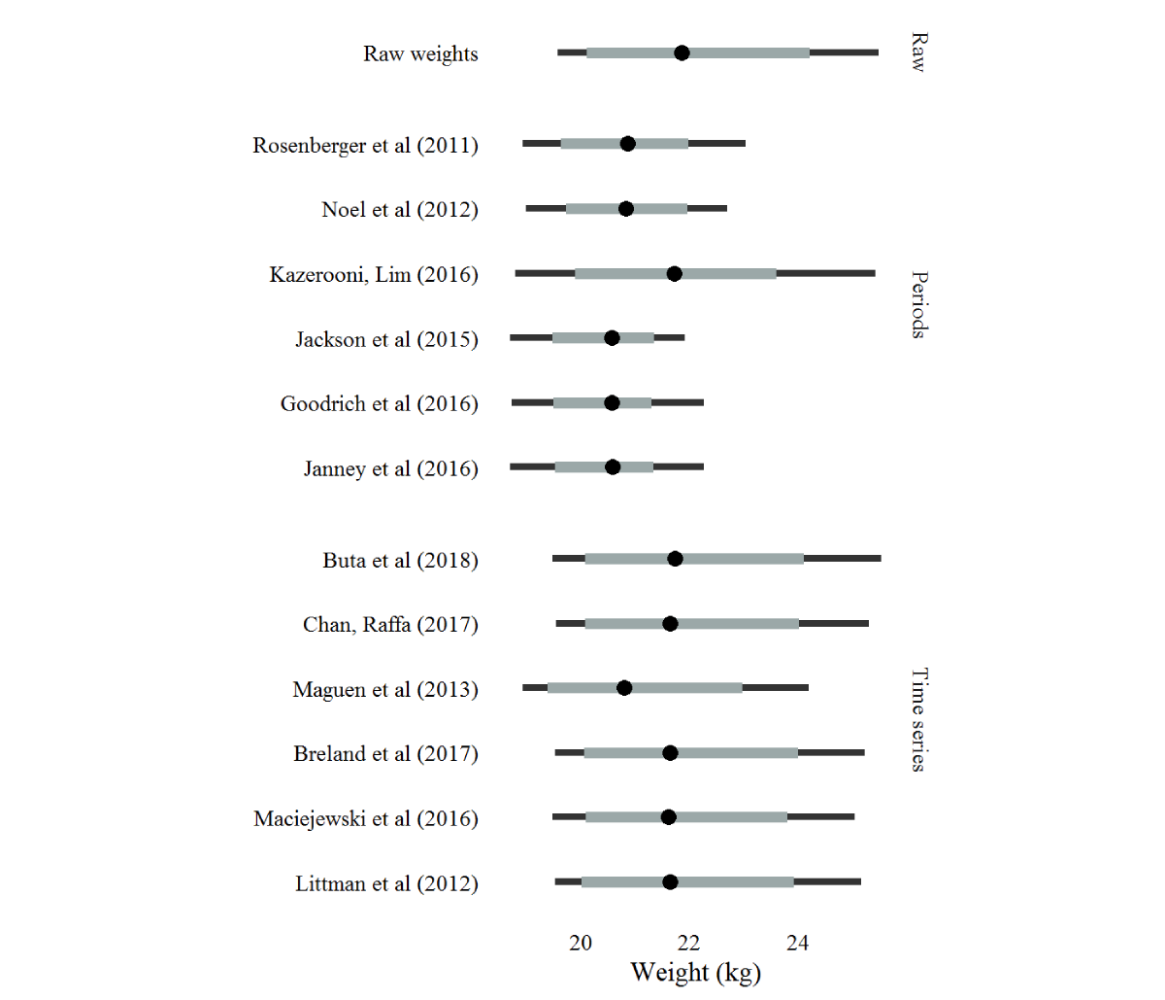
Bootstrapped 95% CI of the SD by algorithm and algorithm type. The midpoint represents the median SD, the thick gray line represents the 80% quantile interval, and the black line represents the 95% quantile interval [23-25,30,35,36,38,39,41-43,46].

Although the mean weight did not change appreciably between the 12 algorithms, there were noticeable differences in the resulting distributions of weight. To explore these differences, we implemented a bootstrap procedure for the mean and variance by sampling 1000 patients, with replacement—thus each patient could be in each sampling iteration more than once—then evaluating the sample data with all 12 algorithms, and repeating this procedure 100 times. Each algorithm is designed to *clean* weight measurements; thus, in terms of the mean, the differences between algorithms are minute (Multimedia Appendix 1, Figure S1), rarely deviating from the mean of the unprocessed data. Differences in variance stand in stark contrast, deviating in both measures of center and spread between algorithms and years—most notably, Kazerooni and Lim [23] and Maguen et al [26] (Figure 1 [17-28]). Disregarding the standout algorithms, differences in SD were still small on an absolute scale, with an approximate range between algorithms of 0.9 kg and 1.8 kg.

### Algorithms Applied to Analysis Scenarios

#### Weight as a Predictor

**Figure 2:**
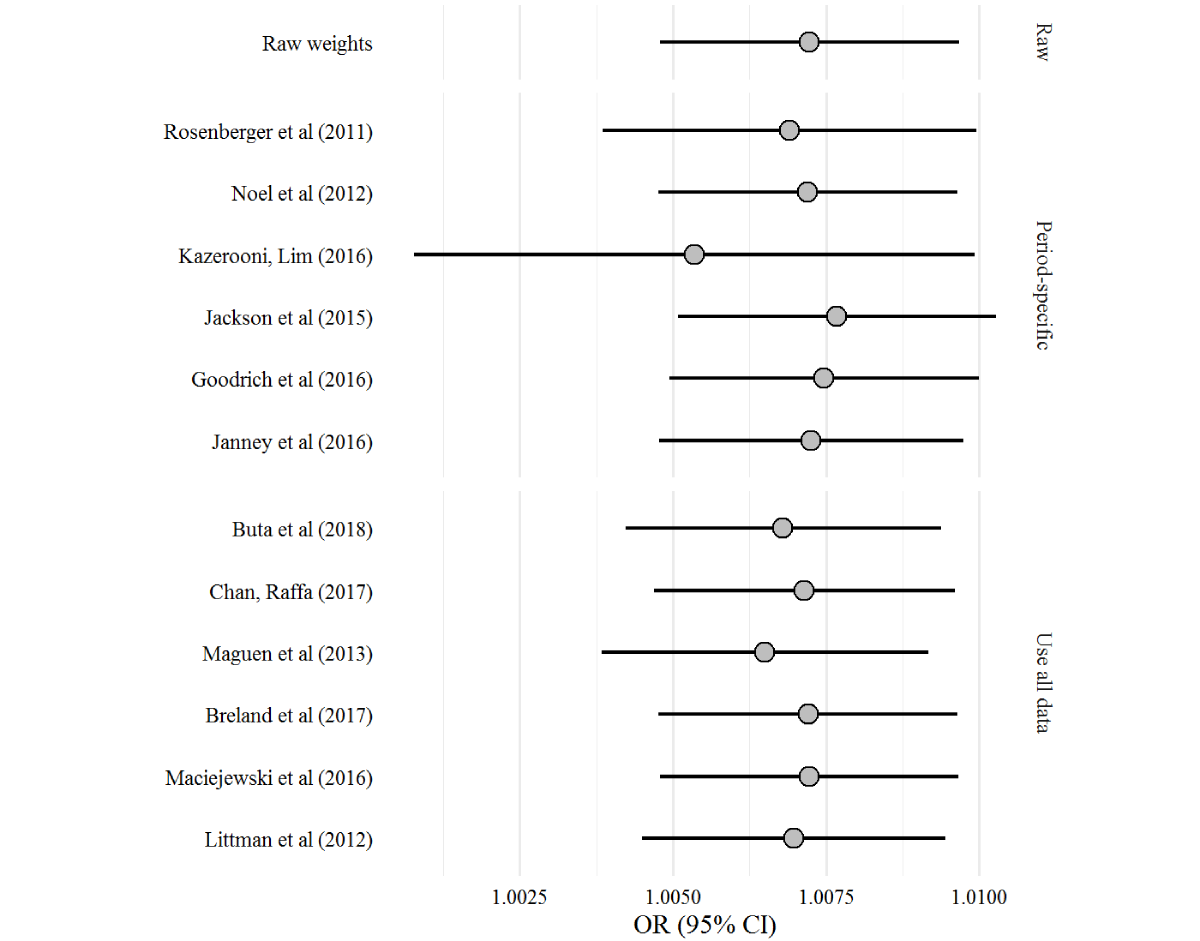
Odds ratio (OR; 95% CI) from 13 separate logistic regressions predicting new-onset diabetes as a function of weight [23-25,30,35,36,38,39,41-43,46].

A total of 13 individual logistic regressions were computed to predict the occurrence of new-onset diabetes as a function of weight. The reported OR and 95% CI varied little between algorithms, and all ORs were slightly >1.00 (Figure 2 [17-28]). The results from the Kazerooni and Lim [23] algorithm are the most striking, exhibiting the widest CI and the smallest OR (see the web-based supplement section *Weight as a Predictor* for a detailed exploration of each analytic decision [50]).

#### Weight Change (Gain and Loss)

Table 3 shows descriptive statistics for ≥5% weight loss and gain by algorithm. To calculate 1-year weight loss and gain, patients were required to have both a baseline weight (60 days before to 60 days after the index date) and a follow-up weight (60 days before to 60 days after 1 year from the index date). After applying the algorithms, only 24% (23,987/99,958) to 60.25% (60,225/99,958) of patients were retained for analysis and, unsurprisingly, the algorithms that used all data retained the most patients. However, the proportion of patients with ≥5% weight loss remained stationary at roughly 13.13% (7851/59,773) to 13.95% (5425/38,875) for nearly all algorithms. The exception was the Maguen et al [26] algorithm, which resulted in only 9.37% (4933/52,642) of patients achieving this weight loss goal. A similar pattern in the results is exhibited by the weight gain analysis—Maguen et al [26] resulted in the lowest gain (4088/52,642, 7.77%), whereas all others stayed relatively the same at 10.86% (6494/59,770) to 12.15% (4725/38,875).

The average weight change was slightly <0, ranging from −0.13 kg to −0.43 kg, with the largest discrepancy resulting from the Maguen et al [26] algorithm and the smallest from the Kazerooni and Lim [23] algorithm. Despite the often lengthy processing steps involved in each algorithm, almost all algorithms still retained implausible weight change outliers, ranging from −1454 kg to −242 kg for the Rosenberger et al [28] and Kazerooni and Lim [23] algorithms, respectively (see Multimedia Appendix 1, Figure S2 for a graphical representation).

**Table 3:** Comparing weight loss metrics by algorithm, common measures of weight loss ≥5%, and average weight change from baseline.

Item		Patients retained^a^, n (%)	Weight loss ≥5% from baseline, n (%)	Weight gain ≥5% from baseline, n (%)	Average weight change from baseline (kg), mean (SD; range)
Raw weights		60,286 (60.3)	8162 (13.5)	6977 (11.6)	−0.13 (7.3; −456 to –485)
**Algorithms that used all data**					
	Buta et al [18]	57,014 (57)	7762 (13.6)	6642 (11.6)	−0.27 (5.4; −111 to –126)
	Chan and Raffa [19]	60,175 (60.2)	8069 (13.4)	6902 (11.5)	−0.26 (5.4; −231 to –126)
	Maguen et al [26]	52,642 (52.7)	4933 (9.4)	4088 (7.8)	−0.17 (3.5; −33 to –44)
	Breland et al [17]	60,225 (60.3)	8124 (13.5)	6936 (11.5)	−0.27 (5.2; −117 to –94)
	Maciejewski et al [25]	58,457 (58.5)	7985 (13.7)	6810 (11.6)	−0.28 (5.1; −53 to –88)
	Littman et al [24]	59,773 (59.8)	7851 (13.1)	6787 (11.4)	−0.22 (4.9; −54 to –49)
**Period-specific algorithms**					
	Rosenberger et al [28]	38,875 (38.9)	5425 (14)	4725 (12.2)	−0.31 (6.4; −454 to –135)
	Kazerooni and Lim [23]	23,987 (24)	3355 (14)	2503 (10.4)	−0.43 (5.6; −242 to –136)
	Goodrich et al [20]	58,142 (58.2)	7828 (13.5)	6688 (11.5)	−0.27 (5.2; −53 to –93)
	Janney et al [22]	58,171 (58.2)	7842 (13.5)	6679 (11.5)	−0.28 (5.4; −132 to –127)
	Jackson et al [21]	59,770 (59.8)	7973 (13.3)	6494 (10.9)	−0.32 (5.1; −111 to –104)
	Noël et al [27]	58,525 (58.5)	7786 (13.3)	6624 (11.3)	−0.26 (5.2; −111 to –88)

^a^Number of patients retained after applying the algorithm. N=99,958 (number of veterans in the 2016 cohort).

#### Weight Trajectory

For each algorithm, the individual trajectories were modeled using a random slope and intercept. Latent class membership represents a choice by the statistical modeler; here, for both conceptual and parsimonious reasons, a 3-class model was chosen for analysis.

**Figure 3:**
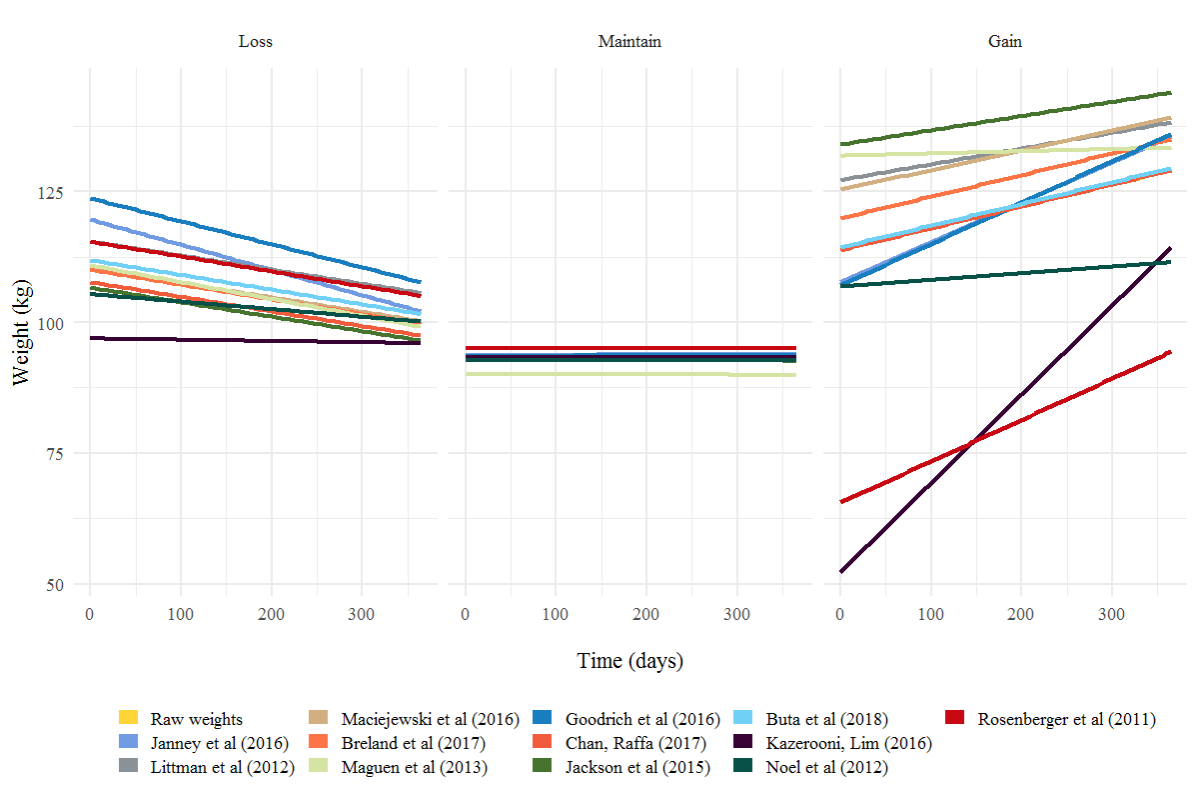
Group-based trajectory modeling by algorithm [23-25,30,35,36,38,39,41-43,46].

Figure 3 [17-28] displays predictions from latent class LMMs computed for each algorithm. There are three types of trajectories: those displayed with a negative slope (predicted weight loss), a slope of nearly 0 (corresponding to those predicted to maintain weight across a 1-year span), and a positive slope (predicted weight gain).

The choice of algorithm can affect predicted weight loss and weight gain within 1 year. Each algorithm produced a slightly different slope and intercept for each class (eg, for the raw data, *β*_1,_*_t_*=–0.00906 vs *β*_2,_*_t_*=.00000 and *β*_3,_*_t_*=.01322 for classes 1, 2, and 3, respectively), implying that the second class of individuals maintained their weight over time, whereas class 1 was predicted to lose 1.5 kg, and class 3 was predicted to gain 2.2 kg over a period of 365 days. For all but 1 algorithm, the posterior probability of individuals classified as class 1 (loss) was low, with a median across algorithms of 0.34 (range 0.014-0.99; Multimedia Appendix 1, Table S13), implying that 3.4% (34/1000) of sampled veterans were predicted to lose weight. The Kazerooni and Lim [23] algorithm differed and classified 99.6% (262/263) of its patients as class 1 (loss), with almost 0% predicted for class 2 (maintenance). Goodrich et al [20], Janney et al [22], Kazerooni and Lim [23], and Rosenberger et al [28] stand out with the steepest slopes in class 3 (gain), indicating greater predicted weight gain for patients in this class.

#### Facility-Level Metrics

**Figure 4:**
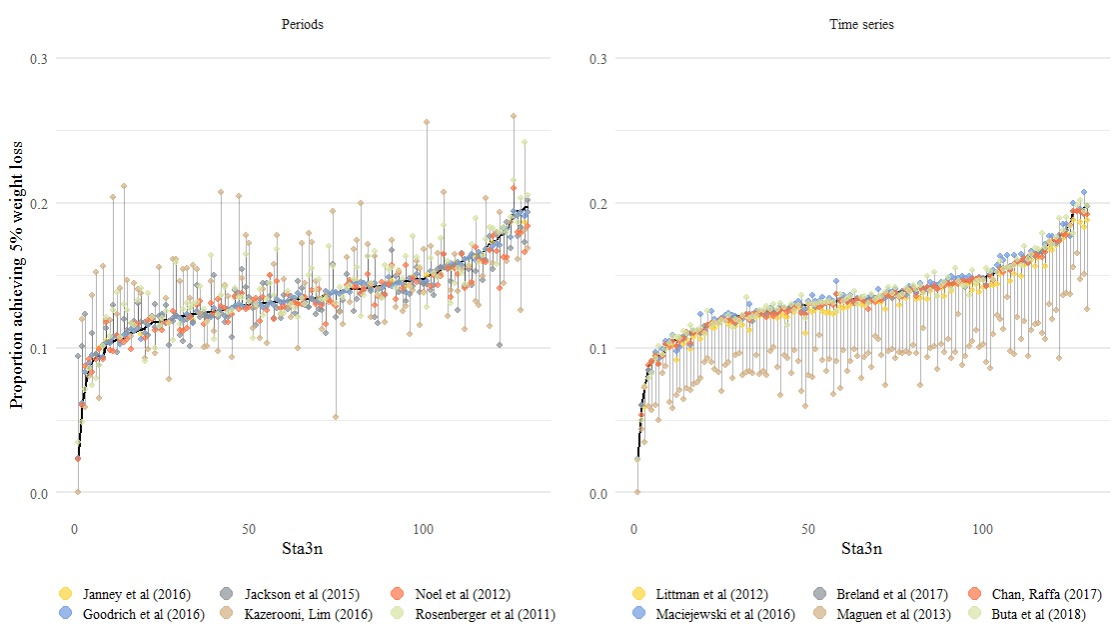
Facility-level percentage of patients with ≥5% weight loss by algorithm. Facilities are ranked along the x-axis according to the percentage of patients who met the metric using raw data, with higher-ranking facilities having greater rates of patients meeting the metric. The percentage of patients who met the metric calculated by each algorithm is displayed for each facility [23-25,30,35,36,38,39,41-43,46].

The percentage of patients with ≥5% weight loss and gain was calculated for each of the 130 facilities using the raw weight data and the weight data as processed by each algorithm. Using the raw data, the percentage of patients with ≥5% weight loss ranged from 2% (1/44) to 19.7% (78/395) across facilities, with an average of 13.5% (SD 2.6%). Across algorithms, the percentage of patients who met the metric ranged from a minimum of 2% (1/44) to a maximum of 26% (13/50). For weight gain, the percentage of patients with ≥5% weight gain ranged from 6% (14/234) to 20% (9/44) across facilities using the raw data, with an average of 11.6% (SD 2.3%); across algorithms, the percentage of patients ranged from 3.1% (12/386) to 27% (14/51). Figure 4 [17-28] shows the facility-level rates, with facilities ranked along the x-axis according to the percentage of patients who met the metric using raw data. Higher-ranking facilities had greater rates of patients meeting the metric. Using the period-specific algorithms to define the percentage of patients with ≥5% weight loss resulted in more variability, and the choice of algorithm clearly affected facility rank. In contrast, the algorithms that used all data exhibited similar ranking to the raw data. The Maguen et al [26] algorithm was a clear outlier and resulted in much lower rates that would affect facility ranking. The Maciejewski et al [25] algorithm showed slightly higher rates.

## Discussion

### Principal Findings

For many applications, the differences between weight-processing algorithms are minor, implying that a simpler algorithm design may be accurate and computationally more efficient in many scenarios. Furthermore, in some cases, the results are not appreciably different from using raw, unprocessed data.

There are subtleties between each algorithm and algorithm type that appear to be more appropriate for specific applications. For example, if it is assumed within a cohort that weight will be lost or gained linearly (eg, weight loss programs or patients with terminal cancer), the Maguen et al [26] algorithm would be appropriate to use.

Studies using point estimates of weight (descriptive statistics and weight as a predictor) and weight change may benefit from a simple cleaning rule based on cutoffs of implausible values, such as excluding weights <34 kg or >318 kg. However, we also recommend examining the computed *weight change* (output) for implausible values in addition to filtering the unprocessed measurements.

Among the algorithms that used all weight measures, most removed outliers within patients, often using some variation of *rolling* SDs to determine implausible values. However, the results from the study by Buta et al [18] are consistent with these algorithms even though the algorithms simply apply an outlier filter based on BMI to the entire sample.

Studies examining weight trajectories and facility-level metrics may benefit from a more nuanced algorithm that considers all available weight data. With respect to trajectory analyses, Kazerooni and Lim [23] and Janney et al [22], both period-specific algorithms, showed steeper weight losses and thus inconsistent results compared with other algorithms. Clearly, when modeling trajectories, the estimation would benefit from using an algorithm that uses all available weight data. In terms of facility-level analysis, all period-specific algorithms resulted in inconsistent or noisy results in comparison with the algorithms that used all data. The clear exception was the Maguen et al [26] algorithm, which *assumes* linearity in weight over time when cleaning weight measurements, an assumption that may not be tenable.

As an example of a recommendation, based on preliminary findings, we used a 2-stage algorithm to derive and clean a weight outcome for the study by Miech et al [52], specifically ≥5% weight loss in a 1-year time frame. The procedure used to arrive at the final outcome was as follows: for each patient in the VHA-derived cohort, all weight data were collected between a patient’s *baseline* time point and the end of follow-up (1 year). To clean these data, the Breland et al [17] algorithm was used as it uses all data, shows consistent results in comparison with other algorithms that use all data, and provides a reasonable distribution of weight values upon computing weight change. Alternatively, the Maciejewski et al [25] algorithm could have been chosen as it exhibits the same ideal characteristics as the Breland et al [17] algorithm yet comes with added complexity in terms of parameter settings because of its design expectant of large changes in weight. Once cleaned with the Breland et al [17] algorithm, weight change and weight change as a percentage of body weight were calculated, and implausible values left in this distribution were then assessed iteratively by choosing the next closest measurement to either the baseline or follow-up weight and then re-examining the weight change distribution. This process ended when the distribution was removed of all implausible values given a range chosen by the study investigators.

### Considerations

These data can be stratified in many ways and, for the purposes of brevity, we chose to display the results assuming homogeneity of the sample. Alternatively, stratifying by demographic or clinical factors had the potential to change our results and conclusions; thus, we chose to differentiate our analysis for patient sex and for categories of weight—namely, underweight, overweight, and obese (web-based supplement [50]). For the sex subanalysis, the patterns of postalgorithm measurements did not differ between men and women save for the noisy facility-level analysis, which can be attributed to the small number of women in multiple facilities. A similar result can be seen in the analysis by BMI category, where the patterns were similar, but the facility-level analysis was noisy because of small numbers. Consequently, the value in further subanalyses should be explored to better address common clinical and research scenarios.

Similar to the choice of data, the methods we chose to address the impact of algorithms were tested on a small selection of analytic approaches while disregarding others that researchers may wish to use. Chiefly, we did not examine the impact on a broader set of machine learning or artificial or computational intelligence approaches common in big data analytics. Further combining machine learning, missing data imputation, and the impact of algorithm choice could prove to be an invaluable resource for the clinical research community.

### Limitations

Our data lack a gold standard and thus, we cannot establish that a presumed outlier is in fact implausible; it is possible that some individuals experienced drastic weight changes that were not considered. Patients who were pregnant during the period were excluded; however, other diseases or conditions may be associated with dramatic weight shifts, and amputation in diabetic patients could also be considered. We did examine the impact of including weight measures from the inpatient setting as well as bariatric surgery patients and found only 2 individuals with implausible weight change values (web-based supplement [50]).

In addition, many algorithms were designed using a specific cohort of patients or an analytic approach, which may not transfer to a general patient cohort. The Maciejewski et al [25] algorithm was designed specifically for studies involving patients who had undergone bariatric surgery or patients who experienced drastic weight changes within a short amount of time. Furthermore, Noël et al [27] proceeded by aggregating longitudinally measured weight over fiscal quarters, a method more appropriate for econometric-type research studies.

Our conclusion that applying a simple algorithm or filter may be enough to *clean* the data has been arrived at by analyzing large samples; thus, these results may differ in smaller samples or small subpopulations, as can be seen in the sex and BMI category analyses. We did not analyze the differences in the algorithms because of the sample size in this study. A simulation study would be warranted to fully assess the impact of sample size.

Finally, all algorithms were reconstructed from the published methods and supplemental material, and there was potential for misinterpretation. In the era of big data analytics and use of patient EHR data for research and evaluation, it is essential that details surrounding data processing and measure creation are included in supplemental materials or shared code (eg, GitHub, Bitbucket, or Docker) to facilitate reproducibility and replication efforts.

### Conclusions

In this paper, we presented several applications of algorithms to process weight measurements obtained from EHRs and attempted to provide recommendations for common research scenarios. Different algorithms result in generally similar results. In some cases, the results are not different from using raw, unprocessed data, despite algorithm complexity. Studies using point estimates of weight (descriptive statistics and weight as a predictor) and weight change may benefit from a simple cleaning rule based on cutoffs of implausible values. Research questions involving weight trajectories and facility-level metrics may benefit from a more nuanced algorithm that considers all available weight data.

## Appendix

Multimedia Appendix 1 Supplementary tables and figures.

## Abbreviations

CDW: Corporate Data Warehouse
EHR: electronic health record
LMM: linear mixed model
OR: odds ratio
VHA: Veterans Health Administration
