# Supplementary material for: Deriving Weight From Big Data: Comparison of Body Weight Measurement–Cleaning Algorithms
Source: JMIR Med Inform. 2022 Mar 9;10(3):e30328. doi: 10.2196/30328 (PMC8943548; doi:10.2196/30328)
Supplement: Multimedia Appendix 1 [file medinform_v10i3e30328_app1.docx]

**Supplementary Materials for:** *Deriving Weight from Big Data: A Comparison of Body Weight Measurement Cleaning Algorithms*

Richard Evans, MS^1^, Jennifer A. Burns, MHSA^1^, Ann Annis, RN, PhD^1,2^, Michelle B. Freitag MPH^1^, Susan D. Raffa PhD^3,4^, Laura J. Damschroder, MS, MPH^1^, Wyndy L. Wiitala, PhD^1^

^1^Center for Clinical Management Research, VA Ann Arbor Healthcare System, Ann Arbor, MI

^2^College of Nursing, Michigan State University, East Lansing, MI

^3^National Center for Health Promotion and Disease Prevention, Veterans Health Administration, Durham, NC

^4^Department of Psychiatry & Behavioral Sciences, Duke University School of Medicine, Durham, NC

Online Supplement Available Here: <https://github.com/CCMRcodes/WeightAlgorithms>

1. **Sample**
   1. **Sample Collection**

Data collection proceeded in two stages, first SQL Server using the CDWWork Database through the VHA CDW. The full procedure is as follows,

1. Collect veterans with at least 1 PCP visit in 2008
   1. Include Age ≥ 18
   2. Select 1st PCP visit in 2008 (stop code 323)
   3. Randomly select 100,000 Patients
   4. Collect all weights from 2 years prior to 2 years post 1st PCP visit (index visit)
2. Collect veterans with at least 1 PCP visit in 2016
   1. Include Age ≥ 18
   2. Select 1st PCP visit in 2016
   3. Randomly select 100,000 Patients
   4. Collect all weights from 2 years prior to 2 years post 1st PCP visit
3. Collect Height Samples from 2 years prior to years post the index visit.
   - 1. **Special Considerations**

We sought to identify subpopulations that may not be representative of a typical cohort drawn for research and evaluation purposes. First, we excluded 1,516 women who were pregnant at any time within the 4-year window (e.g., for a PCP visit in 2008, between 2 years prior and 2 years-post PCP visit). Diagnosis codes to identify pregnancy are lengthy and can be found in the online supplement <https://github.com/CCMRcodes/WeightAlgorithms/tree/master/ClinicalCodes>

We excluded 1,219 patients who received bariatric surgery within the 4-year window, as their weight trajectories over time can be irregular, in fact, the **Maciejewski *et al.*** algorithm was designed specifically for processing patient weights following gastric bypass surgery. These 1,219 patients contributed 44,359 weight measurements across all cohorts.

*Table 1.* ICD/CPT Codes Defining Bariatric Surgery

| Procedure | Code Type | Code |
| --- | --- | --- |
| RYGB, Open | ICD-10 | 0D1607A, 0D160ZA, 0D1607B, 0D160ZB |
|  | CPT | 43633, 43846, 43847 |
| RYGB, Laparoscopic | ICD-10 | 0D1647A, 0D1647B, 0D164ZA, 0D163ZA, 0D1687A, 0D1687B, 0D168ZA, 0D168ZB |
|  | CPT | 43644, 43645, 43844, S2085 |
| AGB, LAGB | ICD-10 | 0DV60CZ, 0DV63CZ, 0DV64CZ |
|  | CPT | 43770, 43843, S2082 |
| Sleeve | ICD-10 | 0DB64Z3, 0DB60Z3, 0DB63Z3, 0DB67Z3, 0DB68Z3 |
|  | CPT | 43775 |

We also identified weights collected during inpatient stays for use in sensitivity analyses. Across all four cohorts there were 2,845 Veterans with an inpatient stay, contributing 23,118 weight measurements.

- 1. **Sample Exploration**
     1. **Overall**

*Table 2.* Overall Sample Size by Year

| Sample Year | N |
| --- | --- |
| 2008 | 98786 |
| 2016 | 98958 |

*Table 3.* Number of Weight Measurements per Person

| Sample Year | mean | SD | median | min | max |
| --- | --- | --- | --- | --- | --- |
| 2008 | 12.29 | 15.97 | 9 | 1 | 1479 |
| 2016 | 12.21 | 24.85 | 8 | 1 | 4981 |

*Table 4.* Number of Height Measurements per Person

| Sample Year | mean | SD | median | min | max |
| --- | --- | --- | --- | --- | --- |
| 2008 | 5.60 | 5.33 | 4 | 1 | 131 |
| 2016 | 5.53 | 5.00 | 4 | 1 | 105 |

- - 1. **Age Distribution**

*Table 5.* Age (At Index Visit) Distribution by Sample Year

| Sample Year | mean | SD | median | min | max |
| --- | --- | --- | --- | --- | --- |
| 2008 | 64.04 | 14.80 | 63.77 | 18.18 | 101.09 |
| 2016 | 62.95 | 15.80 | 66.19 | 19.05 | 104.15 |

- - 1. **Race**

*Table 6.* Number of Weight Measurements by Race and Sample Year

|  | Sample Year | |
| --- | --- | --- |
| Race | 2008 | 2016 |
| White | 870,225 (71.7%) | 868,279 (71.8%) |
| Black or African American | 207,869 (17.1%) | 237,615 (19.7%) |
| Native Hawaiian or Other Pacific Islander | 11,344 (0.9%) | 10,755 (0.9%) |
| American Indian or Alaska Native | 8,299 (0.7%) | 10,000 (0.8%) |
| Asian | 6,473 (0.5%) | 11,327 (0.9%) |
| Multi-Racial | 8,020 (0.7%) | 6,636 (0.5%) |
| Missing | 102,163 (8.4%) | 64,122 (5.3%) |

*Table 7.* Number of Patients by Race and Sample Year

|  | Sample Year | |
| --- | --- | --- |
| Race | 2008 | 2016 |
| White | 71,269 (72.1%) | 73,200 (74.0%) |
| Black or African American | 13,501 (13.7%) | 16,178 (16.3%) |
| Native Hawaiian or Other Pacific Islander | 860 (0.9%) | 897 (0.9%) |
| American Indian or Alaska Native | 610 (0.6%) | 856 (0.9%) |
| Asian | 601 (0.6%) | 947 (1.0%) |
| Multi-Racial | 544 (0.6%) | 479 (0.5%) |
| Missing | 11,401 (11.5%) | 6,401 (6.5%) |

- - 1. **Sex**

*Table 8.* Number of Weight Samples by Sex and Sample Year

| Sample Year | Sex | N (%) |
| --- | --- | --- |
| 2008 | Male | 1,147,194 (94.5%) |
|  | Female | 67,199 (5.5%) |
| 2016 | Male | 1,128,782 (93.4%) |
|  | Female | 79,952 (6.6%) |

*Table 9.* Number of Patients by Sex and Sample Year

| Sample Year | Sex | N (%) |
| --- | --- | --- |
| 2008 | Male | 93,937 (95.1%) |
|  | Female | 4,849 (4.9%) |
| 2016 | Male | 92,758 (93.7%) |
|  | Female | 6,200 (6.3%) |

- - 1. **Bariatric Surgery Patients**

*Table 10.* Number of Bariatric Surgery Patients by Sample Year

| Sample Year | N (% of Denominator) |
| --- | --- |
| 2008 | 15 (0.02) |
| 2016 | 36 (0.04) |

- - 1. **Inpatient Weights**

*Table 11.* Number of Inpatients Identified

| Sample Year | Inpt./Outpt. | N (%) |
| --- | --- | --- |
| 2008 | Inpatient | 282 (0.28) |
|  | Outpatient | 98,783 (99.72) |
| 2016 | Inpatient | 567 (0.57) |
|  | Outpatient | 98,957 (99.43) |

*Table 12.* Number of Inpatient vs. Outpatient Weights Identified

| Sample Year | Inpt./Outpt. | N (%) |
| --- | --- | --- |
| 2008 | Inpatient | 3,224 (0.27) |
|  | Outpatient | 1,211,169 (99.73) |
| 2016 | Inpatient | 4,638 (0.38) |
|  | Outpatient | 1,204,096 (99.62) |

- - 1. **Weight and BMI Distributions**

*Table 13.* Distribution of Collected Weight Data, by Sample Year

| Sample Year | N (%) | Mean | SD |
| --- | --- | --- | --- |
| 2008 | 1,214,393 (17.7) | 202.66 | 47.65 |
| 2016 | 1,208,734 (17.7) | 207.57 | 48.55 |

*Table 14.* Distribution of Computed BMI, by Sample Year

| Sample Year | N (% of Collected Weight) | Mean | SD |
| --- | --- | --- | --- |
| 2008 | 1,116,234 (91.9) | 29.34 | 6.86 |
| 2016 | 1,095,555 (90.6) | 29.93 | 7.64 |

1. **Algorithm Design**

All R and SAS code created to analyze each algorithm available in the online supplement.

- 1. **Janney *et al.* 2016**
     1. **Excerpt from Published Methods**

“Baseline, 6- and 12-mo body weight measures were retrieved for each MOVE! veteran from the VHA patient care databases. Baseline weight was measured within 30 d of MOVE! enrollment. The closest weight within a 60-d window of the follow-up target date (180 d for 6 mo. and 365 d for 12 mo.) was selected/defined as weight for 6- and 12-mo. follow-ups. Weight was coded missing if not available at baseline, 6 mo., and/or 12 mo. Outliers were defined as baseline weight less than 91 lb. or greater than 600 lb..; 6- or 12-mo weight less than 72 lb. or greater than 650 lb.; weight change from baseline greater than 100 lb. …”

- - 1. **Translation in Pseudocode**

| **Algorithm: Janney 2016** |
| --- |
| DEFINE time t_ij for person i IN 1:I, j weights IN 1:J {0mo., 6mo., 12mo.}  FOR i IN 1:I  weight_i1 := weight @ t_i1 +/- 30 days (baseline)  IF (weight_i1 < 91 lbs. OR weight_i1 > 600 lbs.)  weight_i1 := NA  FOR j IN 2:J  weight_ij := weight @ t_ij +/- 60 days  IF (weight_ij < 72 lbs. OR weight_ij > 650 lbs.)  weight_ij := NA  END FOR  END FOR |

- 1. **Littman *et al.* 2012**
     1. **Excerpt from Published Methods**

“For weight, height, and body mass index, we first removed biologically implausible values (weight <75 lb. or >600 lb., height <49 in or >94 in, and body mass index >80 kg/m.). Next, we applied algorithms to identify measures that were plausible but appeared to be erroneous on the basis of a review of all recorded weights and heights during the relevant time period. After reviewing records that had large standard deviations (SDs) (explained in more detail below), we used the algorithm that follows to exclude values that were likely erroneous while keeping values that were plausible. We excluded any weight measurements that met the following 2 criteria: 1) the difference between the mean weight and weight in question was greater than the SD and 2) the SD was greater than 10% of the mean. For example, 1 participant’s weight in pounds was recorded as 300 and 160 lb., both measured on December 7, 2005, 310 lb. measured on June 12, 2006, 276 lb. measured on August 8, 2006, 291 lb. measured on August 15, 2006, and 291 lb. measured on September 13, 2007, resulting in mean (SD) of 271.3 (55.7) lb. The weight of 160 lb. recorded on December 7, 2005, was considered erroneous and dropped because the difference between the index weight and mean weight was greater than the SD (271 − 160 = 113.3 lb.) and the SD was greater than 10% of the mean of all weights ([55.7/271.3] × 100 = 20.5%).”

- - 1. **Translation in Pseudocode**

| **Algorithm: Littman 2012** |
| --- |
| DEFINE person i IN 1:I, j date IN 1:J, weight k IN 1:K (additional recorded weight, same day) |
| FOR i IN 1:I |
| FOR j IN 1:J |
| FOR k IN 1:K |
| weight_ijk := weight for ith person on jth date and kth weight |
| END FOR |
| END FOR |
| IF (weight_ijk < 75 lbs OR weight_ijk > 600 lbs) |
| weight_ijk := NA |
| MEAN_i := MEAN({weight_i}_jk) |
| SD_i := SD({weight_i}_jk) |
| IF (ABS(MEAN_i - weight_ijk) > SD_i AND SD_i > 0.10 * MEAN_i) |
| weight_ijk := NA |
| END FOR |

- 1. **Maciejewski *et al.* 2016**
     1. **Excerpt from Published Methods**

“…Among the [cases], approximately 3.5% of weights were measured on the same day. If the standard deviation of the same-day weights was less than or equal to 2 lb, then the mean was taken. Otherwise, the standard deviation of each same-day weight with prior/post weight measurements was calculated and the same-day weight leading to the smallest standard deviation was retained. After sorting weight measures by date for each individual, rolling standard deviations were calculated using consecutive groups of three weight measures for each individual. The first group consisted of weight measures 1-3, the second 2-4, and so forth. The first and last groups were evaluated separately because the first (and last) weight measure could only be included in one group and the second (and next to last) could only be included in two groups. If the first (or last) two groups’ standard deviations were greater than 35 lb., the second (or next to last) weight measure was deleted. If the first (or last) standard deviation was greater than 35 lb., then paired standard deviations were calculated for each pair within the first (or last) group. If two of the paired standard deviations were greater than 45 lb. and the remaining was less than 10 lb., the offending weight measure was deleted. After these deletions, the weight measures were reassembled in date order for each individual and rolling standard deviations were recalculated and assigned to the central weight measure of the group. Clusters of high standard deviations, indicating a potential outlier, were identified by flagging consecutive standard deviations greater than 10 lb. For each cluster of high standard deviations of three or more the interior weight measures were deleted keeping only the first and last measures of the cluster. Approximately 1.2% of weight measurements were identified as outliers and were deleted. Standard deviation cutoffs were determined via iterative trial and error driven by clinical plausibility of the specific measure rather than a standard rule and with guidance from clinical practitioners familiar with context of surgery and expected outcomes. Before the same-day and outlier cleaning, the cohort … had 89,757 measurements; after these cleaning steps, 85,556 (95.3%) weight measurements remained.”

There is an extra step not mentioned in the methods but detailed elsewhere. All weights must first be between 50 and 700 lbs. (inclusive), then different weights recorded on the same day are dealt with as described above.

Batch *et al*. (2018) used this same algorithm in their paper Outcome by Gender in the Veterans Health Administration Motivating Overweight/Obese Veterans Everywhere Weight Management Program.

- - 1. **Translation in Pseudocode**

| **Algorithm: Maciejewski 2016** |
| --- |
| DEFINE person i IN 1:I, j dates IN 1:J, weight measurements k IN 1:K |
| FOR (i IN 1:I) |
| FOR (j IN 1:J) |
| FOR (k IN 1:K) |
| weight_ijk := weight for ith person, jth date and kth measurement |
| END FOR |
| END FOR |
| #TODO: Finish this - the way this is documented in the methods is confusing |
| END FOR |

- 1. **Breland *et al.* 2017**
     1. **Excerpt from Published Methods**

From Supplementary Materials 3a. Creating clean weight values:

1. We searched for specific patterns likely to be correct weights incorrectly entered by human error (e.g., a numeric value followed by pound, lb, ll, l, le, kilo, kg, k, etc.). Weight values matching these patterns were corrected (e.g., 300L became 300 or 50kg became 110). N = 27,550 records.
2. “Unavailable” and other non-numeric values were set to missing (e.g., what appeared to be a date entered in the weight field). N = 485,962 records.
3. We rounded all weight values to the nearest hundredth pound.
4. We set outlier weights to missing (<75 pounds or >700 pounds). N = 141,688 records.
5. We identified and set to missing weights representing unlikely weight trajectories:
   1. Created Ratio 1 variable: current weight/prior weight
   2. Created Ratio 2 variable: current weight/next weight
   3. Created an indicator variable (R1_ind) using Ratio 1:
      1. If Ratio 1 <= 0.67 then R1_ind = -1;
      2. If Ratio 1>= 1.50 then R1_ind = 1;
      3. R1_ind = 0 for all other values of Ratio 1
   4. Created an indicator variable (R2_ind) using Ratio 2:
      1. If Ratio 2 <= 0.67 then R2_ind = -1;
      2. If Ratio 2>= 1.50 then R2_ind = 1;
      3. R2_ind = 0 for all other values of Ratio 2
   5. Weights were set to missing if (R1_ind = 1 AND R2_ind = 1) OR (R1_ind = -1 AND R2_ind = -1). N = 254,481 records.
6. We removed missing weight records. N = 882,131 weight records; N = 590,143 patients. Removing these records resulted in removing 121,299 patients from the dataset as they had no valid weight records.
   - 1. **Translation in Pseudocode**

| **Algorithm: Breland 2017** |
| --- |
| DEFINE t_j, weight_j in 1:J |
| IF (type_of(weight)) == CHAR |
| IF (weight contains extraneous non-numeric values) |
| weight := RECODE(weight) |
| weight := CAST(weight, NUMERIC) |
| weight := ROUND(weight, 2) |
| IF (weight == NA OR weight == 'unavailable') |
| weight := NA |
| IF (weight < 75 lbs. OR weight > 700 lbs) |
| weight := NA |
| DEFINE Ratio1 as (current weight):(prior weight) |
| DEFINE Ratio2 as (current weight):(next weight) |
| weight_{0} := weight_0 (initialize to baseline weight) |
| FOR j IN J |
| Ratio1 := weight_j / weight_{j - 1} |
| IF (Ratio1 <= 0.67) |
| I(Ratio1) := -1 |
| ELSE IF (Ratio1 >= 1.50) |
| I(Ratio1) := 1 |
| ELSE I(Ratio1) := 0 |
| Ratio2 := weight_j / weight_{j + 1} |
| IF (Ratio2 <= 0.67) |
| I(Ratio2) := -1 |
| ELSE IF (Ratio2) >= 1.50 |
| I(Ratio2) := 1 |
| ELSE I(Ratio2) := 0 |
| IF (I(Ratio1) = 1 AND I(Ratio2) = 1) OR (I(Ratio1) = -1 AND I(Ratio2) = -1) |
| weight_j := NA |
| END FOR |

- 1. **Maguen *et al.* 2013**
     1. **Excerpt from Published Methods**

“…For our analysis, we used BMI measurements up to 3 years following the index weight measurement (after the last deployment); the average patient-level BMI was calculated for each 6-month interval starting at the index BMI measurement. Only biologically plausible heights and weights were included in the analyses (> 70 lb. and < 700 lb.; > 46 in. and < 84 in.). To further improve data quality, all available weight measurements (both pre-deployment and post-deployment) were included in a linear mixed model of BMI over time (with a random intercept and slope for each veteran, adjusted for age and gender), in order to identify and exclude within-patient outliers (absolute value of conditional residual ≥ 10). BMI measurements were retained if they were both biologically plausible and not extreme outliers (16 ≤ BMI ≤ 52).”

- - 1. **Translation in Pseudocode**

| **Algorithm: Maguen 2013** |
| --- |
| DEFINE time t_ij for person i IN 1:I, j weights IN 1:J |
| FOR i IN 1:I |
| FOR j IN 1:J |
| IF (70 lb. <= weight_ij <= 700 lb.) |
| weight_ij := weight @ t_ij |
| END FOR |
| Compute LMM per person: |
| Weight_ij := (beta_0 + veteran_effect) + (beta_1 + veteran_effect) * time_j + beta_2 * age + beta_3 * gender + e_ij |
| IF (abs({Estimated Weight_i} \| t, age, gender) >= 10) |
| Weight_ij := NA |
| END FOR |

- 1. **Goodrich *et al.* 2016**
     1. **Excerpt from Published Methods**

“…cohort criteria required participants to have a baseline weight documented within 1 month before or after MOVE! enrollment (index date) and at least one follow-up weight at 6 or 12 months after enrollment…Participant records with implausible values for weights (<80 lb. or >500 lb.) and heights (<48 inches or >84 inches) were excluded, as were those with implausible 6- and 12-month weight changes (>100 lb.)”

- - 1. **Translation in Pseudocode**

| **Algorithm: Goodrich 2016** |
| --- |
| DEFINE time t_ij for person i IN 1:I, j weights IN 1:J {0mo., 6mo., 12mo.} |
| FOR i IN 1:I |
| weight_i1 := weight @ t_i1 +/- 30 days (baseline) |
| IF (weight_i1 == NA) |
| EXCLUDE person i |
| IF (weight_i1 <= 80 lbs. OR weight_i1 >= 500 lbs.) |
| EXCLUDE person i |
| FOR j IN 2:J |
| weight_ij := weight @ t_ij +/- 60 days |
| IF (weight_ij <= 80 lbs. OR weight_ij >= 500 lbs.) |
| EXCLUDE person i |
| END FOR |
| IF (abs(weight_{i, j+1} - weight_{ij}) > 100 lbs.) |
| EXCLUDE person i |
| END FOR |

- 1. **Chan & Raffa, 2017**
     1. **Excerpt from Published Methods**

“A three-step cleaning process was used to identify outlier weights recorded in the EHR. The first step was to exclude 70,636 (0.27%) weights in the EHR with values less than 22.7 kg (50 lbs.) or greater than 340.2 kg (750 lbs.), as these extreme values are implausible. The second step was to calculate each Veteran’s BMI using the average height, in order to identify the 45,318 weights that were similarly implausible (defined as a BMI ≤10 or BMI ≥100) for exclusion. Then, for the third step, each Veteran’s mean weight and standard deviation were determined from the remaining weights available. Weights in the EHR that were greater than 3 standard deviations from the mean, an additional 162,349 weights, were excluded. Overall, following the three-step cleaning procedure, a total of 278,303 (1.05%) weights were excluded from the analysis leaving 26,263,946 weights available for inclusion.”

- - 1. **Translation in Pseudocode**

| **Algorithm: Chan & Raffa 2017** |
| --- |
| DEFINE time t_ij for person i IN 1:I, j weights IN 1:J |
| FOR i IN 1:I |
| FOR j IN 1:J |
| IF (weight_ij < 50 lbs. OR weight_ij > 750 lbs.) |
| weight_ij := NA |
| END |
| MEAN_i := MEAN({weight_i}_j) |
| SD_i := SD({weight_i}_j) |
| FOR j IN 1:J |
| IF (weight_ij > MEAN_i +/- 3 * SD_i) |
| weight_ij := NA |
| END FOR |
| END FOR |

- 1. **Jackson *et al.* 2015**
     1. **Excerpt from Published Methods**

“BMI was assessed using clinically recorded weight and height, after excluding implausible values (approximately 0.1%)…Weight was recorded as the patient’s baseline weight, and follow-up weights as average weight within subsequent time windows (6 mo.: 3–9 mo.; 12 mo.: 9–15 mo.; 24 mo.: 21–27 mo.; 36 mo.: 33–39 mo.).”

- - 1. **Translation in Pseudocode**

| **Algorithm: Jackson 2015** |
| --- |
| DEFINE time t_ijk for person i IN 1:I, j weights IN 1:J {0mo., 6mo., 12 mo., 24mo., 36mo.},  k measurments IN 1:K |
| FOR i IN 1:I |
| weight_i1 := weight @ t_i1 (baseline) |
| FOR j IN 2:J |
| FOR k IN 1:K |
| weight_ijk := all k weights between t_ij +/- 90 days |
| END FOR |
| IF (weight_ij < 75 lbs OR weight_ij > 700 lbs.) |
| weight_ij := NA |
| weight_ij := MEAN({weight_ijk}) |
| END FOR |
| END FOR |

- 1. **Buta *et al.* 2018**
     1. **Excerpt from Published Methods**

“For each participant, we used VA electronic health record (EHR) data to obtain all BMI measurements available from the date of their first medical visit through September 30, 2010. Because we were interested in BMI change over time, we excluded participants who had no or only one BMI measurement available (260,193 participants left) … BMI was computed based on height and weight data routinely collected and recorded in EMR records during VA clinical visits. We removed from analyses a small percentage (0.03%) of biologically implausible BMI values (BMI<11 or BMI>70).”

- - 1. **Translation in Pseudocode**

| **Algorithm: Buta 2018** |
| --- |
| DEFINE time t_ij for person i IN 1:I, j BMI IN 1:J |
| FOR i IN 1:I |
| FOR j IN 1:J |
| BMI_ij := BMI @ t_ij |
| END FOR |
| Let k = Number of Non-missing j BMI for person i |
| IF (k <= 1) |
| EXCLUDE person i |
| END FOR |

- 1. **Kazerooni & Lim, 2016**
     1. **Excerpt from Published Methods**

“Patients were excluded from this study if they were missing weight values from any of the three measuring points of the study (pre-, mid-, and post-weight) … Pre-weight was defined as the most recent weight within 30 days before starting topiramate therapy. Mid-study period was defined as the earliest weight taken between 3 and 6-months post initiation of therapy. Post-study period was defined as the earliest weight taken between 6 and 12-months post initiation of therapy.”

- - 1. **Translation in Pseudocode**

| **Algorithm: Kazerooni & Lim, 2016** |
| --- |
| DEFINE time t_ij for person i IN 1:I, j weights In 1:J {pre-trt, mid-trt, post-trt} |
| FOR i IN 1:I |
| FOR j IN 1:J |
| weight_i1 := weight @ t_0 (treatment start) - 30 days |
| weight_i2 := {t_0 + 90 days <= weight <= t_0 + 180 days} |
| weight_i3 := {t_0 + 180 days < weight <= t_0 + 365 days} |
| END FOR |
| IF (weight_ij IS NULL) (weight measure missing at jth time point) |
| EXCLUDE person i |
| END FOR |

- 1. **Noel *et al.* 2012**
     1. **Excerpt from Published Methods**

“… we used BMI derived from heights and weights obtained during routine clinical encounters. These data are stored in facility information systems and uploaded into the Corporate Data Warehouse; validation work indicates that some of these height and weight values probably reflect data entry errors. Therefore, we used an iterative process to eliminate or control for height and weight outliers while avoiding suspect BMI values. In specifying the original cohort of obese primary care patients, we removed biologically “implausible” values (i.e., 70 lbs. > weight or weight > 700 lbs. and 48 in. > height or height > 84 in.). We then divided each of the five study years into quarters and determined the median value for weights recorded during each quarter for every patient, yielding up to 20 quarterly median weights.”

- - 1. **Translation in Pseudocode**

| **Algorithm: Noel 2012** |
| --- |
| DEFINE weight_ij for person i IN 1:I and weight j @ time t |
| Divide time vector into q Fiscal Quarters |
| FOR i IN 1:I |
| FOR j IN 1:J |
| IF (weight_ij > 70 lbs. AND weight_ij < 700 lbs.) |
| weight_ij := weight @ t_ij |
| END FOR |
| FOR q IN 1:Q |
| weight_iq := MEDIAN({weight_i}_j) (all weights within quarter q) |
| END FOR |
| END FOR |

- 1. **Rosenberger *et al.* 2011**
     1. **Excerpt from Published Methods**

“To obtain our study sample, starting from the date of the first VHA healthcare visit for each veteran, we calculated BMI at VHA visits within a six month time frame for up to 6 years. Thus, while all veterans had different actual starting dates, time in the study began for each veteran at their first VHA healthcare visit. Each veteran had at most 12 BMI observations. A missing BMI value was generated if weight was not recorded during a specific 6-month window. We limited our analytic sample to veterans who had at least seven observed BMI values after their last deployment end date. This restriction was necessary to collect sufficient and necessary information for determining the BMI trajectories.”

- - 1. **Translation in Pseudocode**

| **Algorithm: Rosenberger 2011** |
| --- |
| DEFINE t_ij for person i IN 1:I, weight j IN 1:J {0 (baseline): 6 years, by 0.5 years} |
| FOR i IN 1:I |
| FOR j IN 1:J |
| weight_ij := weight @ t_ij +/- 180 days |
| // (every 6 months starting from t_0 = baseline) |
| END FOR |
| IF (COUNT({weight_i}_j) < 7 |
| EXCLUDE person i |
| END FOR |

1. **Algorithm Comparison**
   1. **By Algorithm, and Sample Year**

*Table 15.* Weight Processing by Algorithm and Type of Algorithm – PCP 2016 Cohort

| Weight Algorithm | N pts. Retained  (% of Raw) | N Weights Retained  (% of Raw) | Mean  (SD) | Median  (IQR) | Range  (Min, Max) |
| --- | --- | --- | --- | --- | --- |
| Raw Weights | 98,958 (100) | 1,208,734 (100) | 208 (49) | 202 (61) | (0, 1486) |
|  |  |  |  |  |  |
| Utilize All Data |  |  |  |  |  |
| Buta (2018) | 90,444 (91) | 1,165,326 (96) | 208 (48) | 202 (61) | (60, 540) |
| Chan (2017) | 96,367 (97) | 1,202,912 (99) | 208 (48) | 202 (61) | (54, 728) |
| Maguen (2013) | 98,391 (99) | 1,067,245 (88) | 205 (46) | 200 (59) | (70, 541) |
| Breland (2017) | 98,958 (100) | 1,207,922 (99) | 208 (48) | 202 (61) | (75, 694) |
| Maciejewski (2016) | 98,958 (100) | 1,144,843 (94) | 208 (48) | 203 (60) | (62, 546) |
| Littman (2012) | 96,365 (97) | 1,194,256 (99) | 208 (48) | 202 (60) | (75, 546) |
|  |  |  |  |  |  |
| Time-Period Specific |  |  |  |  |  |
| Rosenberger (2011) | 63,405 (64) | 227,215 (19) | 208 (46) | 203 (58) | (0, 1315) |
| Kazerooni (2016) | 23,987 (24) | 71,961 (6) | 209 (48) | 204 (60) | (0, 1234) |
| Goodrich (2016) | 95,749 (97) | 208,790 (17) | 206 (45) | 201 (57) | (80, 500) |
| Janney (2016) | 95,743 (97) | 208,829 (17) | 206 (45) | 201 (57) | (75, 546) |
| Jackson (2015)* | 96,559 (98) | 251,501 (21) | 206 (46) | 201 (57) | (76, 553) |
| Noel (2012)* | 98,958 (100) | 683,008 (57) | 207 (46) | 202 (58) | (70, 589) |

***** These algorithms differ from the other time-period specific algorithms as they first use all available data, then proceed to aggregate measures by the mean or median within select time-periods.

*Figure 1.* Bootstrapped mean and 95% CI of mean weight by algorithm and cohort.

*
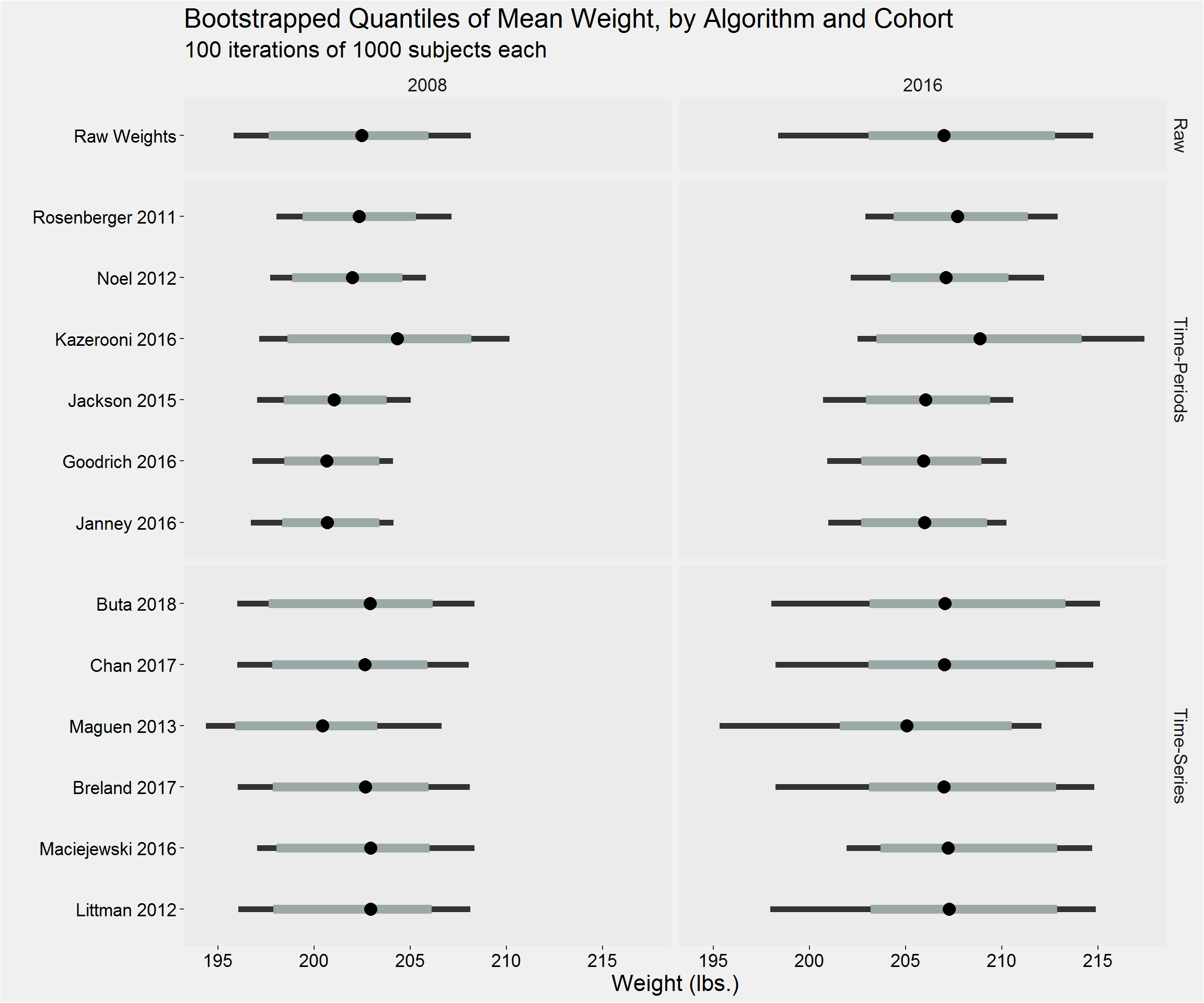
*

- 1. **Weight Loss Comparison**

The probability of classification into class 1 or class 2 can be examined from each algorithm: probability of class 1 membership (negative slope) was low, with a mean across algorithms of 4.2% (range: 2.1-7.3%; Supplementary Table 13), implying that approximately 4% of MOVE! 2016 participants are predicted to lose weight. As a measure of model performance, we can examine the conditional probabilities of class membership, for example, the probability of belonging to class 1, given that the model has classified the patient as class 1, is 0.93, while the probability of belonging to class 2 given that the model has classified an individual as class 1 is 0.0057. Similarly, the probability of being categorized in class 1 given that the LCMM categorized a specific patient as class 2 is 0.0665, and the probability of being in class 2 given that an individual is class 2 is 0.9943. These conditional probabilities imply that the model predicted class membership quite well. The same conclusion can be reached looking across weight cleaning algorithms. Note how the probability of class 1 membership has decreased for the Janney, Jackson, and Goodrich algorithms in comparison with the others (and increased in improper prediction into class 2).

*Figure 2.* Weight Change Outliers, Post Algorithm Processing


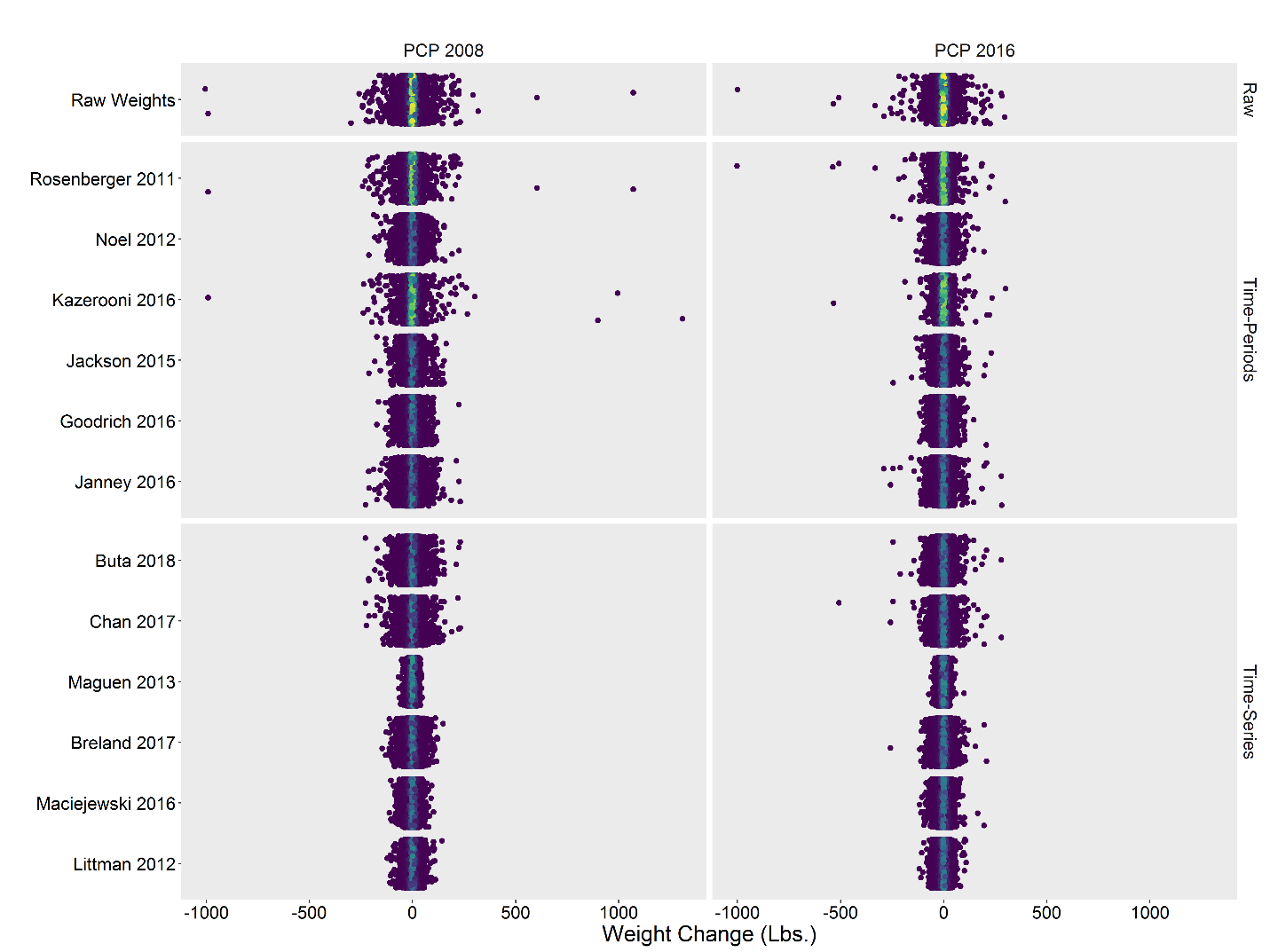


- 1. **Latent Trajectory Analysis**

The probability of classification into class 1 or class 2 can be examined from each algorithm: probability of class 1 membership (negative slope) was low, with a mean across algorithms of 4.2% (range: 2.1-7.3%; Supplementary Table 13), implying that approximately 4% of participants are predicted to lose weight. As a measure of model performance, we can examine the conditional probabilities of class membership, for example, the probability of belonging to class 1, given that the model has classified the patient as class 1, is 0.93, while the probability of belonging to class 2 given that the model has classified an individual as class 1 is 0.0057. Similarly, the probability of being categorized in class 1 given that the LCMM categorized a specific patient as class 2 is 0.0665, and the probability of being in class 2 given that an individual is class 2 is 0.9943. These conditional probabilities imply that the model predicted class membership quite well. The same conclusion can be reached looking across weight cleaning algorithms. Note how the probability of class 1 membership has decreased for the Janney, Jackson, and Goodrich algorithms in comparison with the others (and increased in improper prediction into class 2).

*Table 16.* Posterior Classification, by Algorithm

| **Algorithm** | **Concept class** | **N** | **%** | **p(Loss)** | **p(Maintain)** | **p(Gain)** |
| --- | --- | --- | --- | --- | --- | --- |
| Raw Weights | Loss | 33 | 3.32 | 0 | 0 | 0.96 |
| Raw Weights | Maintain | 951 | 95.77 | 0.91 | 0.02 | 0 |
| Raw Weights | Gain | 9 | 0.91 | 0.09 | 0.98 | 0.04 |
| Breland 2017 | Loss | 35 | 3.52 | 0.88 | 0.02 | 0 |
| Breland 2017 | Maintain | 950 | 95.67 | 0 | 0 | 0.97 |
| Breland 2017 | Gain | 8 | 0.81 | 0.12 | 0.98 | 0.03 |
| Buta 2018 | Loss | 33 | 3.63 | 0 | 0 | 0.97 |
| Buta 2018 | Maintain | 868 | 95.38 | 0.9 | 0.02 | 0 |
| Buta 2018 | Gain | 9 | 0.99 | 0.1 | 0.98 | 0.03 |
| Chan 2017 | Loss | 38 | 3.92 | 0 | 0 | 0.96 |
| Chan 2017 | Maintain | 922 | 95.15 | 0.86 | 0.02 | 0 |
| Chan 2017 | Gain | 9 | 0.93 | 0.14 | 0.98 | 0.04 |
| Goodrich 2016 | Loss | 14 | 1.44 | 0 | 0 | 1 |
| Goodrich 2016 | Maintain | 958 | 98.26 | 0.12 | 0.99 | 0 |
| Goodrich 2016 | Gain | 3 | 0.31 | 0.88 | 0.01 | 0 |
| Jackson 2015 | Loss | 26 | 2.66 | 0.14 | 0.97 | 0.05 |
| Jackson 2015 | Maintain | 940 | 96.21 | 0 | 0.01 | 0.95 |
| Jackson 2015 | Gain | 11 | 1.13 | 0.86 | 0.02 | 0 |
| Janney 2016 | Loss | 14 | 1.44 | 0.14 | 0.99 | 0 |
| Janney 2016 | Maintain | 958 | 98.26 | 0 | 0 | 1 |
| Janney 2016 | Gain | 3 | 0.31 | 0.86 | 0.01 | 0 |
| Kazerooni 2016 | Loss | 262 | 99.62 | 0 |  | 0 |
| Kazerooni 2016 | Maintain | 0 | 0 | 1 |  | 0 |
| Kazerooni 2016 | Gain | 1 | 0.38 | 0 |  | 1 |
| Littman 2012 | Loss | 32 | 3.3 | 0 | 0 | 0.99 |
| Littman 2012 | Maintain | 930 | 95.98 | 0.84 | 0.02 | 0 |
| Littman 2012 | Gain | 7 | 0.72 | 0.16 | 0.98 | 0.01 |
| Maciejewski 2016 | Loss | 38 | 3.83 | 0 | 0 | 0.98 |
| Maciejewski 2016 | Maintain | 948 | 95.47 | 0.86 | 0.02 | 0 |
| Maciejewski 2016 | Gain | 7 | 0.7 | 0.14 | 0.98 | 0.02 |
| Maguen 2013 | Loss | 14 | 1.43 | 0.02 | 0.04 | 0.78 |
| Maguen 2013 | Maintain | 902 | 92.13 | 0.12 | 0.96 | 0.22 |
| Maguen 2013 | Gain | 63 | 6.44 | 0.87 | 0 | 0 |
| Noel 2012 | Loss | 46 | 4.6 | 0.18 | 0.95 | 0.18 |
| Noel 2012 | Maintain | 919 | 91.9 | 0 | 0.02 | 0.82 |
| Noel 2012 | Gain | 35 | 3.5 | 0.82 | 0.02 | 0 |
| Rosenberger 2011 | Loss | 21 | 3.2 | 0 | 0 | 1 |
| Rosenberger 2011 | Maintain | 634 | 96.5 | 0.2 | 0.99 | 0 |
| Rosenberger 2011 | Gain | 2 | 0.3 | 0.8 | 0.01 | 0 |
